# Role of Obesity in Female Reproduction

**DOI:** 10.7150/ijms.80189

**Published:** 2023-01-31

**Authors:** Wei Yong, Jiajia Wang, Yan Leng, Lijie Li, Han Wang

**Affiliations:** 1 Center Laboratory of the Fourth Affiliated Hospital, China Medical University, Shenyang 110032, China.; 2 Affiliated Hospital of Changchun University of Chinese Medicine, Changchun 130021, China.; 3 Third Affiliated Clinical Hospital to Changchun University of Chinese Medicine, Changchun 130021, China.; 4 Institute of Chinese Materia Medica, China Academy of Chinese Medical Sciences, Beijing 100700, China.

**Keywords:** obesity, female reproduction, infertility, oocytes, adipokines

## Abstract

Contemporary scientists need no “p value” and “relative risk” statistics to be exquisitely aware of the increasing prevalence of obesity and complications posed by obesity. It is now well recognized that obesity is strongly associated with type 2 diabetes, hypertension, vascular disease, tumors and reproductive disorders. Obese women show lower levels of gonadotropin hormones, reduced fecundity, higher miscarriage rates and poorer outcomes of *in vitro* fertilization, revealing that obesity affects female reproduction. In addition, adipose tissue contains special immune cells and obesity-induced inflammation is a chronic, low-grade inflammatory response. Herein, we mainly review detrimental influences of obesity in the complete process of female reproduction, including hypothalamic-pituitary-ovarian axis, oocyte maturation, embryo and fetal development. In the latter part, we view obesity-induced inflammation and discuss related epigenetic impact on female reproduction.

## Introduction

The obvious but unfortunate trend towards widespread epidemic of obesity has posed a serious threat to public health worldwide. Over the past few decades, the number of overweight and obese individuals has reached alarming levels, with about 40% of adults worldwide now overweight or obese. For example, the prevalence of obesity and overweight was only 7.8% for Chinese adults in 1985, more than half of adults in China are now categorized as overweight or obese 1. Even worsen, by 2030, the prevalence of overweight and obesity might reach 65.3% in adults if this trend would continue 2. Body mass index (BMI) is an important indicator to measure obesity and standard weight (Normal: 18.5 ≤ BMI ≤ 24.9 kg/m^2^; Overweight: 25.0 ≤ BMI ≤ 29.9 kg/m^2^; Obese: BMI ≥30.0 kg/m^2^), and the risk of obesity complications will increase with the rise of BMI 3.

Adipose tissue, the main storage and transport of energy, plays an important role in the regulation of energy balance. Obesity occurs when the body stores too much energy in the form of fat. Considerable evidence has demonstrated that obesity is not only directly harmful to health, but also is strongly related to a number of chronic diseases such as type 2 diabetes, cardiovascular diseases, respiratory diseases and reproductive disorders 4-6. Obesity reduces fertility, with a 2.7-fold increased risk of infertility in women with a BMI of >30 kg/m^2^ and a 25-37% increased risk of miscarriage in pregnant women compared with normal-weight counterparts 7. Obese women also respond less well to infertility treatments and have a higher risk of early miscarriage after *in vitro* fertilization (IVF), with the live-birth rate reduced by 20% 6-9. Despite clinical impacts of obesity on female reproduction have been well characterized, the underlying mechanisms remain to be elucidated. This article aims to review the relationship between obesity and the complete female reproduction including hypothalamic-pituitary-ovarian axis, oocyte maturation, embryo and fetal development, adding obesity-induced inflammation and related epigenetic impact.

## Effects of Obesity on the Hypothalamic-pituitary-ovarian (HPO) Axis

The hypothalamic-pituitary-ovarian (HPO) axis, a complete and coordinated neuroendocrine system, is essential for female reproductive function. The hypothalamus regulates the release of luteinizing hormone (LH) and follicle stimulating hormone (FSH) in the pituitary by secreting gonadotrophin releasing hormone (GnRH), thus controlling gonadal development and secretion of sex hormones. Obese women showed obviously reduced amplitude, mean LH and higher circulating levels of insulin compared with normal-weight women 9, 10. Insulin promoted the synthesis of androgens, which are aromatized into estrogen in response to excess adipose tissue and ultimately cause negative feedback on the HPO axis 11. Mainly produced by white adipose tissue, leptin is related to modifications of reproductive function. It has been reported that leptin affected GnRH pulse neurons and LH secretion by the pituitary 12-14. A randomized trial mimicking the metabolic syndrome of obesity found that there was no significant difference in serum inflammatory signal and endoplasmic reticulum stress markers, revealing that the endocrine disruption and adverse reproductive outcomes caused by obesity may be mediated by HPO axis directly 15. In support of this central mechanism, it has been demonstrated that the significant reduction in natural pregnancy rate of obese mice induced by a high fat diet can be overcome after exogenous gonadotrophin stimulation 16, 17.

## Effects of Obesity on the Oocyte Maturation

In bisexual reproduction, female gametes bear the main accumulation of energy and matter during the later zygote development, and the development female gametes are more specific and complicated. Follicles are the structural basis of female gamete development in higher animals, especially mammals. During follicular development, on the one hand, the germ cell realizes the orderly arrest and restart of development, and completes the maturation of nucleus and cytoplasm of oocyte. On the other hand, follicular cells undergo multiple differentiation and proliferation, and the orderly development of germ cells is regulated while completing its endocrine function. An important feature of the follicular development initiation stage is the growth of oocyte, which determines the embryo potential. The oocyte in primordial follicles was blocked in the prophase of meiosis I and formed primary oocytes. When oocytes grew to certain sizes, they gained the ability to restore meiosis. At this time, oocytes had large nucleuses, highly loose chromatins, and complete nuclear membranes called germinal vesicle (GV). If completely grown oocytes released from follicular inhibition, spontaneous meiotic recovery may happen, which is called germinal vesicle breakdown (GVBD). After GVBD occurs, oocytes completed the first meiosis, homologous chromosomes were separated, and the first body was discharged 18-20. Then, spindles were assembled again and oocytes entered the metaphase II until fertilization. However, oocytes collected from both obese women and obese mice show poor quality, indicating oocytes negatively impact the oocyte maturation 21-24.

### Meiotic maturation

Complete meiotic maturation contains resumption of meiosis and the correct segregation of chromosomes 25. The oocyte at meiotic arrest is characterized by nucleus having a complete nuclear membrane structure called GV. The most important feature of meiotic resumption is GVBD. After GVBD occurs, the oocyte completed first meiosis, homologous chromosomes were separated, and the first polar body was expelled. Subsequently, the spindle reassembled and entered the second meiotic metaphase until fertilization. In high-fat diet induced obesity mouse models, 39.45% of oocytes from obese mice completed GVBD, whereas 89.46% of control oocytes completed this maturational stage, and oocytes of obese mice were smaller than control oocytes as well 26. Similarly, the maturation of obese mice in IVF was delayed 27.

The spindle is the core of chromosome separation, a dynamic machine mainly composed of a large number of longitudinally arranged microtubules, whose job is to collect and sort chromosomes and distribute them to daughter cells as they divide 28. During meiosis I, GVBD occurs and microtubules form bipolar spindles around chromosomes. At the end of meiosis I stage, spindle migrate to the cortex and cortical reorganization begins. After first polar body extrusion, oocytes enter meiosis II stage and spindles quickly form below the first polar body 29. The integrity of spindles determines the correctness of chromosome segregation, and abnormalities in chromosome segregation can lead to aneuploidy, which contributes to early pregnancy loss 30. The polarized light microscopy can be used to detect spindle abnormalities of oocytes, and compared with the normal BMI groups, the odds of oocytes with disarranged spindles and non-aligned chromosomes was dramatically greater in severely obese groups 31-33. Studies in high fat diet (HFD)-induced obesity models correlated with clinical findings in humans. Both percentages of abnormal spindle morphologies and chromosome misalignment were increased evidently in HFD mice oocytes 30. Furthermore, examination of abnormal oocytes in diet-induced obesity (DIO) mice revealed high rates of meiotic aneuploidy characterized by disorganized spindles and chromosomes improperly aligned on the metaphase plate 34.

### Mitochondrial dysfunction

In general, oocyte maturation involves two different levels of maturation: One is the aforementioned resumption of oocyte meiosis during nuclear maturation. Another one is the cytoplasmic maturation of oocyte, which directly determines the fertilization ability of late oocyte and the ability of early embryo development. It bears mentioning that mitochondria, the principal maternally-inherited organelles in the cytoplasm of oocytes, plays an important role in maturation, fertilization and embryonic development of oocytes, with the main function of manufacturing 5'-adenosine triphosphate (ATP) through actions of the tricarboxylic acid (TCA) cycle and oxidative phosphorylation (OXPHOS) 35. Generally, mitochondrial DNA (mtDNA) copy numbers increase over 30-fold from primary oocytes to MII stage oocytes 36. Oocytes with low mtDNA copy numbers are more likely to be damaged than oocytes with high mtDNA copy numbers in mice and human oocytes which are successfully fertilized contain more mtDNA copies than oocytes which are unsuccessfully fertilized, suggesting that the mtDNA copy number in oocyte corrects with oocyte quality 37-40. Another intriguing feature of mitochondria during oocyte maturation is that the membrane potential of mitochondria increased significantly, which is associated with a concomitant increase in OXPHOS, and the lack of membrane potential may cause the decrease of oocyte development potential 41-43. In addition, the increasing of ATP levels plays a critical role during oocyte maturation and oocytes with higher ATP levels showed a better fertilization rate and embryo development than oocytes with lower ATP levels 44-46.

**Figure 1:**
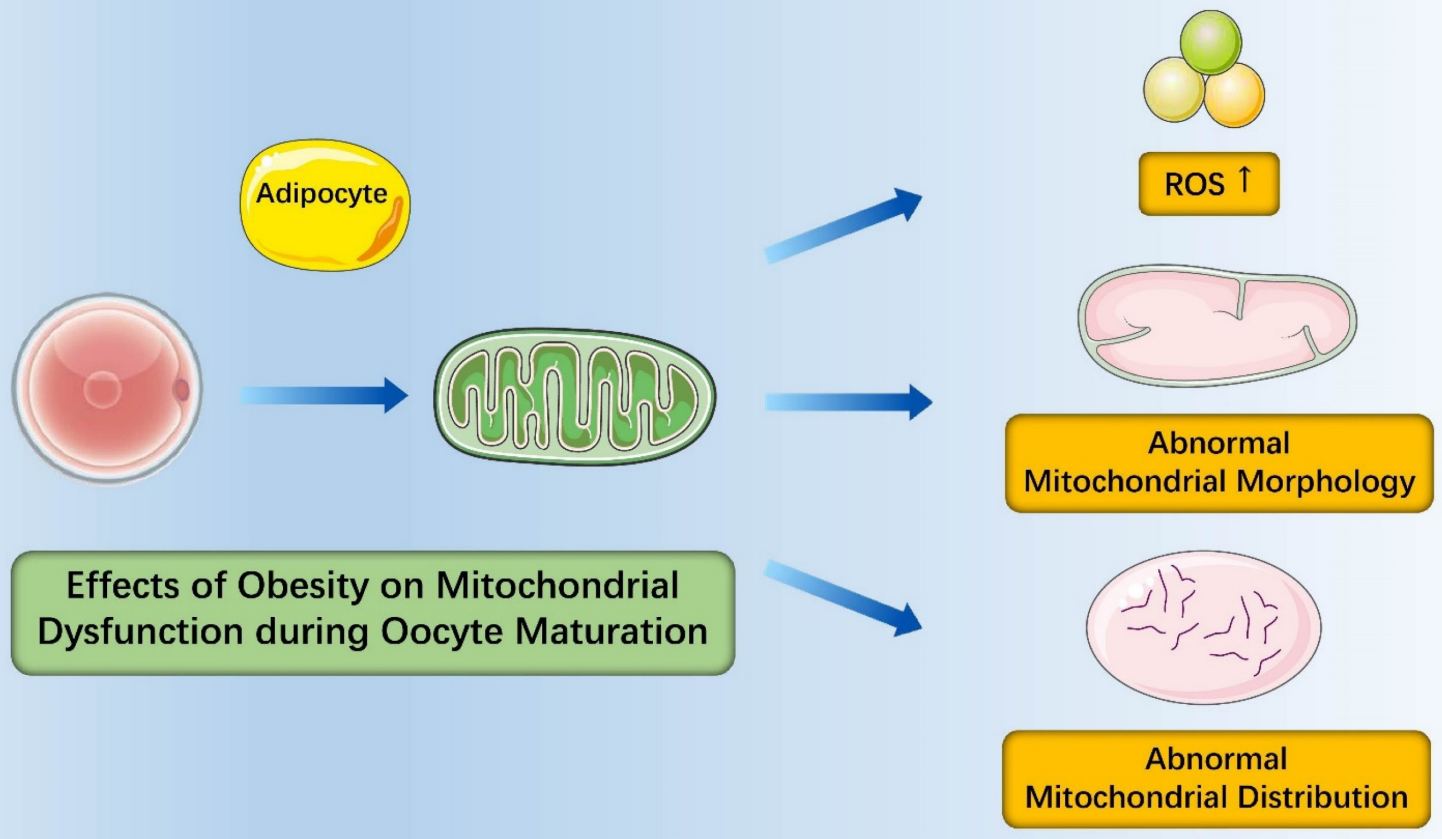
** Effects of obesity on mitochondrial dysfunction during oocyte maturation.** Obesity causes abnormalities of oocyte metabolism with more oxidized oocytes and higher rate of reactive oxygen species (ROS) production; Obesity causes abnormalities of mitochondrial morphology with increased swelling and a growing number of vacuoles; Obesity causes abnormalities of mitochondrial distribution in an unorganized clumping pattern.

In the context of obesity, the number of oocyte mtDNA copies was dramatically higher in obese mice than in those from lean counterparts and the expression of mitochondrial biogenesis and fission markers (PGC-1α and Drp-1) increased in obese mice 34. Similarly, the expression of mtTFAM and NRF1 (nuclear genes encoding mtDNA transcription factors) elevated as well 47. These findings suggested that oxidative stress from obesity induced mitochondrial damage that contributed to compensatory responses of increased mitochondrial biogenesis and fission 35. It is important to note that mitochondria of mice oocytes had fewer cristae which were more disarrayed, decreased electron density of the matrix, increased swelling and a growing number of vacuoles and distribution of mitochondria from obese females was in an unorganized clumping pattern instead of being distributed evenly throughout the ooplasm in mitochondria from normal females 34. Igosheva et al. detected that the membrane potentiala dramatic increased in oocytes and zygotes of C57BL/6J DIO mice by using a potentiometric fluorescent dye with low toxicity 47. Alternatively, most studies found that membrane potential of oocytes mitochondria from obese mice was lower than that of oocytes mitochondria from normal mice 48, 49. To evaluate the redox state of oocytes, studies were performed and found that oocytes from obese mice were more oxidized and had higher rate of reactive oxygen species (ROS) production. Taken together, obesity causes abnormalities of mitochondrial morphology, mitochondrial distribution of oocytes and oocyte metabolism, which ultimately has a negative effect on oocyte maturation (**Figure 1**).

### Endoplasmic reticulum stress

The endoplasmic reticulum (ER) is a cellular organelle playing a critical role in protein biosynthesis, trafficking, folding and Ca^2+^ homeostasis. ER stress occurs when misfolded proteins accumulated in the ER of liver and adipose tissue. Accumulating evidence has revealed that obesity can cause ER stress in mammalian cells 50. ER stress markers inositol requiring enzyme 1α (IRE1α), glucose-related protein 78 (GRP78) and X-box binding protein 1 (XBP1) were significantly increased in adipose tissue of obese compared to lean pregnant women. ER stress was also increased in adipose tissue of women with gestational diabetes mellitus compared to BMI-matched normal glucose tolerant women 51. In placental tissue, transcript levels of ER stress factors XBP1, activating transcription factor 4 (Atf4), and the molecular chaperone calnexin, were decreased with maternal HFD intake 52.

Functional protein synthesis occurs through translation of maternal mRNA and is necessary for oocyte maturation 53-56. During these processes, ER plays significant roles in meeting increased protein demand of oocytes, which is accomplished by correct protein synthesis, folding and modification 57. Therefore, regulation of ER stress is a key mechanism during oocyte maturation. Lipid is deposited not only in adipose tissues, but also in nonadipose tissues, which leads to a high level of free fatty acids and triggering lipotoxicity characterized by ER stress, and ER stress links mitochondrial damage 58. Theres is evidence of ER stress in oocyte that cumulus-oocyte complexes (COCs) treated with thapsigargin (a strong ER stress inducer) decreased cumulus cell expansion (a marker of oocyte quality) and led to poor pre-implantation development rates 59. Similarly, treatment of COCs with palmitic acid (another strong ER stress inducer) decreased cumulus expansion and reduced pre-implantation development 60. To demonstrate that ER stress damages oocyte quality, when cultured with ER stress inhibitor salubrinal, COCs treated with an ER stress inducer or collected from obese mice were surprisingly reversed the oocyte quality by increasing levels of mitochondrial replication factors mitochondrial transcription factor A (TFAM) and dynamin related protein 1 (DRP1) as well as mtDNA in obese mice oocytes 48. Overall, these findings suggest that ER stress plays a crucial role in cumulus-oocyte complex interactions as well as oocyte quality.

## Effects of Obesity on the Embryo

Embryo quality is one of the most critical factors assessing the success of implantation process and subsequent pregnancy 61. In mammals, mature oocytes combine with the sperm to form fertilized eggs. After formation of fertilized eggs, embryos enter the cleavage stage and form solid cell clusters similar to mulberry called morula. As cells divide, fluid-filled cavities appear inside the embryo and cells differentiate into inner cell mass (ICM) and trophoblast cells (TE) 62. The embryo at this stage is called blastocyst. With the development of embryos, they hatch out of the crack in the zona pellucida, and trophoblast cells make contact with the inner wall of the uterus and implant into the uterine tissue, which is called preimplantation embryo development. After implantation, the embryo begins to absorb nutrients in order to maintain development, and gradually differentiates into various cells such as ectoderm, mesoderm and endoderm through gastrula, eventually forming tissues and organs 63-65. To evaluate the association between pregnancy performance and obesity, Zheng et al. used 10,252 frozen-thawed cycles with single blastocyst transfer, and patients were divided into four groups: underweight, normal-weight, overweight, and obesity. Miscarriage rate was higher in the obese group (27.51%), compared with the normal-BMI group (20.91%). Meanwhile, Using the normal-BMI group as reference, the incidence of preterm birth (11.19% vs. 16.87%) and the proportion of women needing a cesarean section (87.91% vs. 93.98%) were higher in the obese group 66. In a retrospective analysis, comparison of human IVF/ICSI cycles with normal BMI groups showed that obesity had adverse effects on the mean embryo grade, the embryo utilization rate and the number of embryos discarded and cryopreserved 67. Similarly, Leary et al. found that embryos from overweight or obese women (BMI ≥25 kg/m^2^) were less likely to accomplish development post-fertilization and more quickly developed to the morula stage with fewer cells in the trophectoderm, reduced glucose consumption, improved amino acid metabolism and increased levels of endogenous triglyceride 68.

As discussed earlier with regard to oocytes, embryos are susceptible to lipotoxicity as well 69, 70. Culturing in excess palmitic acid (PA, the most abundant saturated free fatty acids in human serum and ovarian follicular fluid), murine blastocysts had altered embryonic IGF1 receptor expression, increased glutamic pyruvate transaminase activity and decreased nuclei count. Murine trophoblastic stem cells exposed to PA *in vitro* proliferated less and underwent increased dose-dependent apoptosis. When these embryos transferred into foster mice, fetuses of PA-exposed blastocysts were smaller than controls 68. Apart from this central acting, elevated levels of leptin in obese women exerted a direct negative effect on embryos 71. *In vitro*, leptin stimulated human trophoblastic stem cell growth, and its inhibition modified proliferation and improved apoptosis. Altogether, an obese environment affects embryonic growth and lasted adverse effects on offspring.

## Effects of Obesity on Fetal Development

The placenta, an important organ for the exchange of materials between fetus and mother, is formed by the membrane of the embryo and the endometrium of mother 72. Fetus develops in womb and relies on placenta to obtain nutrients, while maintaining considerable independence 73. Maternal obesity affects both the placenta and the fetus, leading to fetal overgrowth and a higher frequency of large for gestational age (LGA) infants 74. Placental transport was proved to be increased in rodent models of maternal obesity and is closely associated with birth weight of humans 75-77. Children born to obese mothers have higher risks of developing childhood obesity and metabolic disease 78. According to the developmental origins of health and disease (DOHaD) hypothesis, maternal obesity is linked with detrimental cardiometabolic and neurocognitive outcomes in the offspring 79-83. Animal studies have revealed that maternal obesogenic diets induced insulin resistance and increased levels of fetal blood glucose, resulting in accelerated pancreatic β-cell maturation and reduced glucose tolerance in the offspring 84. It has been reported that maternal HFD resulted in decreased number of oocytes and increased apoptosis in fetal ovaries in the rat at Embryonic Day 20 (E20), increased numbers of primordial and transitioning follicles at Postnatal Day 4 (P4), small secondary follicles and increased follicular atresia in prepubertal offspring, and impaired ovarian follicle growth in adult offspring 85. More recent studies have also reported that newborns born to obese mothers had a higher incidence of bacterial and viral infections and required admission to neonatal intensive care units, indicating maternal obesity impacts the fetal immune system 86, 87.

## Inflammation in Female Reproduction with Obesity

**Figure 2:**
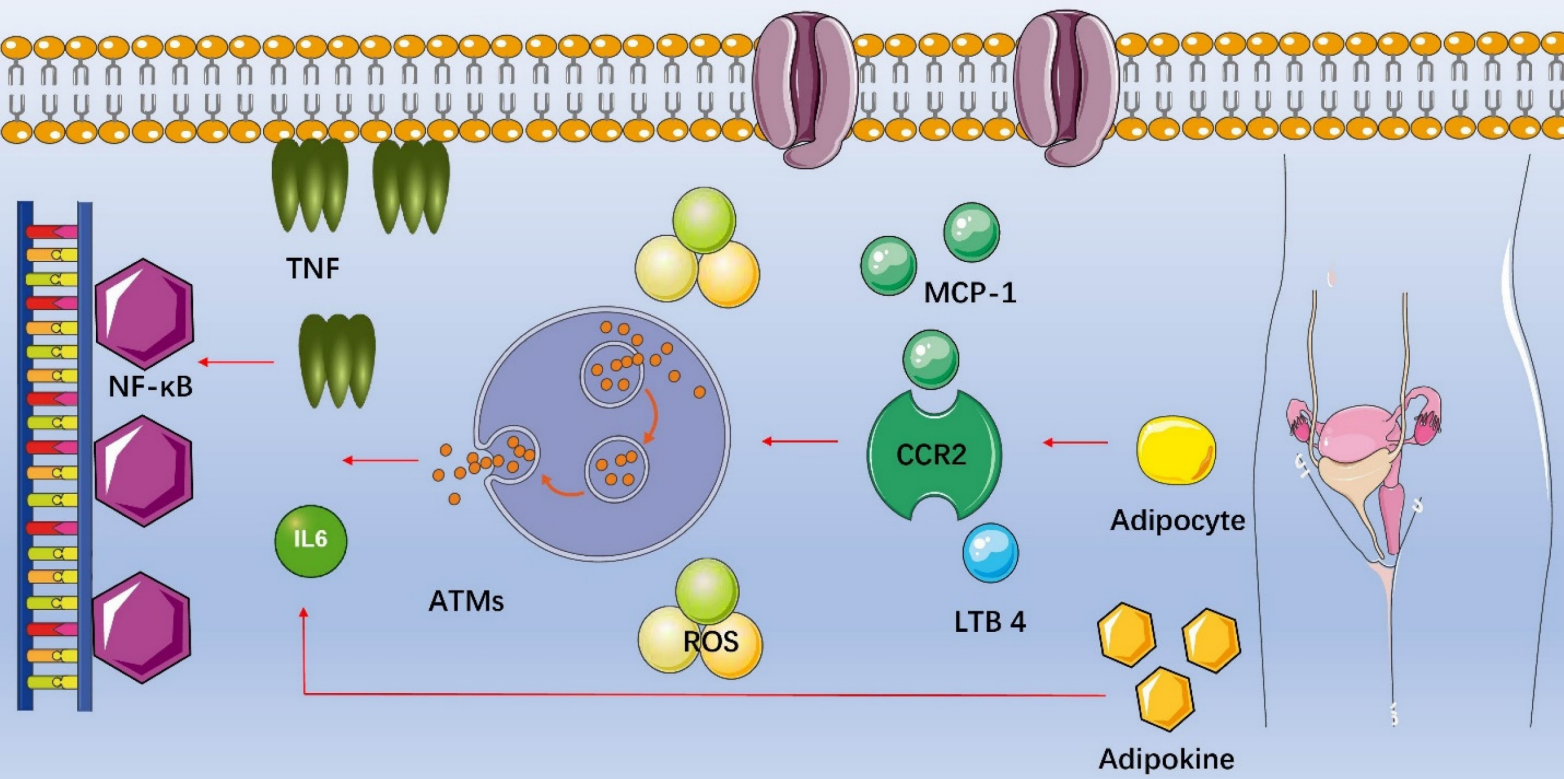
** Inflammation in female reproduction with obesity.** Increasing in circulating triglycerides causes adipocyte secreting chemotactic protein-1 (MCP-1/CCL2), leukotriene B4 (LTB4) or others, attracting monocytes into adipose tissues and becoming adipose tissue macrophages (ATMs). As soon as proinflammatory ATMs move into adipose tissues, they can secrete their own chemokines and attract additional macrophages. Subsequently, macrophages secrete cytokines such as tumor necrosis factor alpha (TNF-α), interleukin-6 (IL-6) and IL-1β, activating the nuclear factor-κB (NF-κB) signal transduction pathway and producing more cytokines. Adipocytes produce adipokines as well, which promotes additional release of TNF-α and IL-6.

Fat is not only a storage organ but also one of the largest endocrine organs in human body, secreting important signaling molecules and expressing a variety of receptors to sense endocrine signals (**Table 1**). Of interest, adipose tissue contains special immune cells and obesity-induced inflammation is a chronic, low-grade inflammatory response caused by excess nutrients in the metabolic cell 88. Macrophages are functionally the most important immune cells in the adipose tissue 89, and the recruitment of macrophages into adipose tissues is the initial event of obesity-induced inflammation. The number of macrophages in adipose tissue increases in obesity and participates in inflammatory pathways activated in fat of obese individuals. Increasing in circulating triglycerides causes adipocyte secreting the chemokine such as monocyte chemotactic protein-1 (MCP-1/CCL2), leukotriene B4 (LTB4) or others, attracting monocytes into adipose tissues and becoming adipose tissue macrophages (ATMs) 90, 91. MCP-1 is a critical chemokine secreted by enlarging adipocytes and stimulates macrophage migration by binding to the C-C chemokine receptor type 2 (CCR2) on macrophages. LTB4, a product of arachidonic acid metabolism, is produced by adipocytes and promotes ATMs infiltration 92. As soon as proinflammatory ATMs move into adipose tissues, they can secrete their own chemokines and attract additional macrophages. In addition, increased secretion of leptin also contributes to macrophage accumulation through stimulating transport of macrophages to fat and adhesion between macrophages and endothelial cells 93. Subsequently, macrophages secrete cytokines such as tumor necrosis factor alpha (TNF-α), interleukin-6 (IL-6) and IL-1β, activating the nuclear factor-κB (NF-κB) signal transduction pathway and producing more cytokines 94. Adipocytes produce adipokines as well, which promotes additional release of TNF-α and IL-6 95 (**Figure 2**). Chronic inflammation induces oxidative stress as well because of increased production of ROS, and ROS are chemically reactive chemicals containing oxygen, mainly including superoxide anion (O_2_^-^), hydroxyl radical (^·^OH) and hydrogen peroxide (H_2_O_2_). Studies showed that excessive ROS in the ovary inhibited the oocyte maturation, decreased the quality of oocytes, caused granular cell (GC) apoptosis and accelerated degeneration of the corpus luteum 96, 97.

There is concern that an altered inflammatory state can impact the growing fetus indirectly by altering a variety of placental functions (e.g., trophoblast invasion or nutrient transport) 98. Aye et al. found that increased maternal BMI was associated with activation of placental p38-MAPK and STAT signaling, demonstrating that inflammation associated with maternal obesity is regulated by altered placental function 99. In addition, gut microbiota differs between obese and normal individuals and plays a role in obesity during female reproduction 100. It has been reported that maternal diet-induced obesity resulted in maternal intestinal infammation, altered fetal glucose metabolism at mid-gestation, and increased risk of metabolic dysfunction in offspring 101.

## The Epigenetic Impact of Obesity on Female Reproduction

All levels of epigenetic regulation seem to have a wide range of effects on development and health 112. For example, epigenetic modification, including acetylation, phosphorylation, methylation, glycosylation and ubiquitination, plays critical roles in the process of female reproduction 113-116. Generally, lysine acetylation of histones is controlled by histone acetyl transferases (HATs) and histone deacetylases (HDACs) and has essential roles in follicle development and oocyte maturation 117. DNA methylation usually involves the addition of a methyl group to the fifth carbon atom of cytosine to form 5-methyl cytosine. Protein phosphorylation occurs most on serine, threonine, or tyrosine residues and regulates cell cycle in various signal transduction pathways 118. The ubiquitination is essential for degradation of proteins, cell cycle process and transcriptional regulation, which is critical for oocyte maturation 119.

However, epigenetic regulation can be altered by diet, BMI, inflammation, oxidative stress and so on 120. In obesity, stable epigenetic changes occur and have detrimental influences during female reproduction 121. It is well established that oxidative stress induces DNA damage reducing the ability of DNA to be methylated by DNA methyltransferases and resulting in global hypomethylation 122. Related factors in obesity can induce epigenetic alterations in adult target cells and epigenetic phenotypes in germline cells that not only impede gamete function, but can also be passed on to the next generation 123. Specific patterns of epigenetic factors were also found to correct with obesity itself in an analysis in leucocytes and adipose tissue 124.

## Conclusion

**Figure 3:**
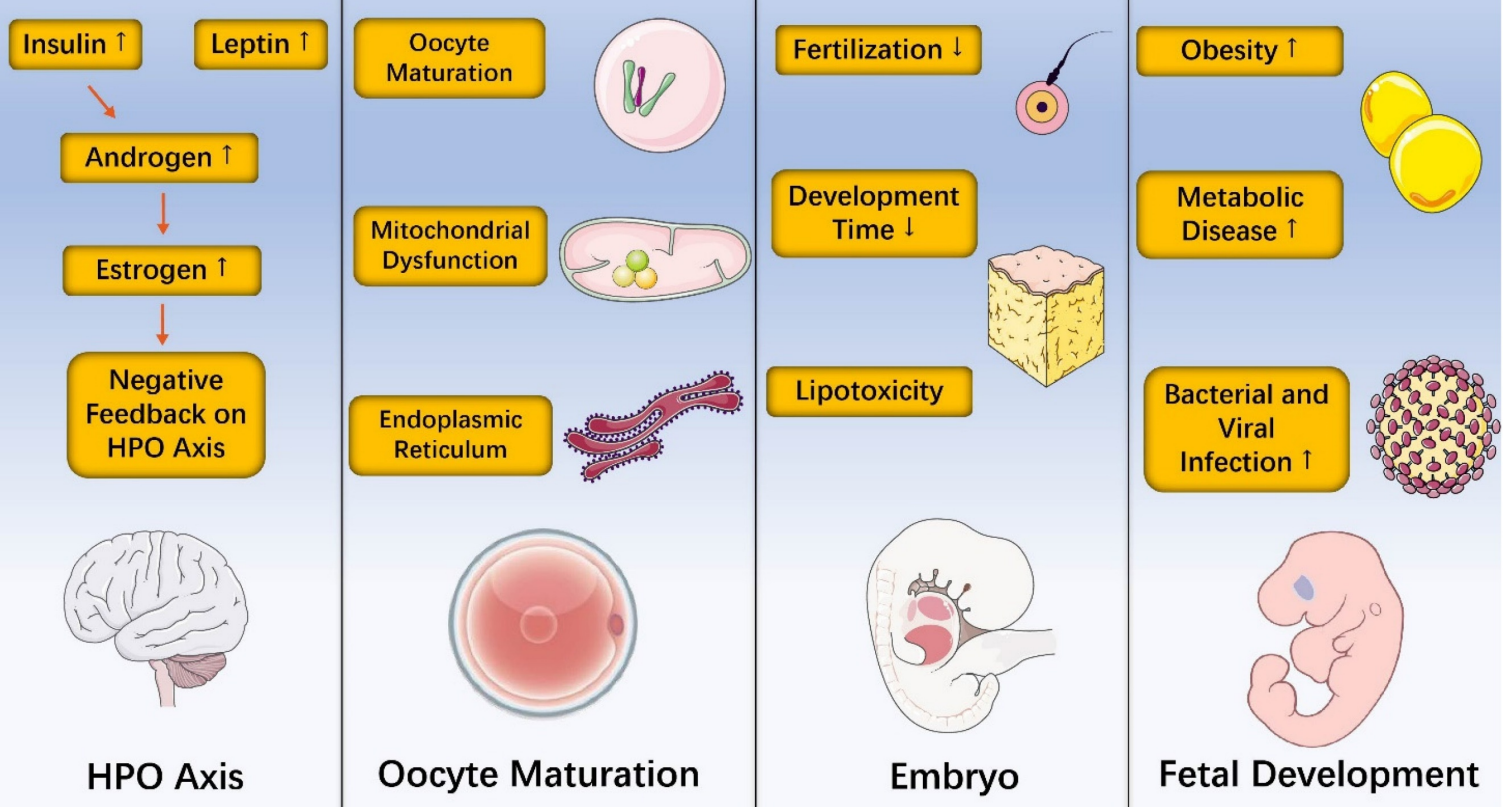
Effects of obesity on hypothalamic-pituitary-ovarian axis, oocyte maturation, embryo and fetal development.

Obesity has become a major public health problem worldwide. It is not only a major risk factor for many common diseases, but also leads directly or indirectly to an increase in health care resources. Compared with lean counterparts, obese women show reduced amplitude, higher circulating levels of insulin, poor oocyte quality, higher miscarriage rates and offspring who are more likely to be sick. In addition, obesity-induced inflammation is a chronic, low-grade inflammatory response and epigenetic changes caused by obesity occur and have detrimental influences during female reproduction. Furthermore, recent data found that losing gut microbes in Drosophila melanogaster suppressed oogenesis 125. This finding, along with observations that polycystic ovarian syndrome (PCOS) was associated with reduced diversity of the gut microbiome in patients from different countries 126-128, collectively support the idea that gut microbiome changes may impact fertility. Taken together, this review discusses effects of obesity on hypothalamic-pituitary-ovarian axis, oocyte maturation, embryo and fetal development (**Figure 3**) with related inflammatory and epigenetic impact. However, more translational work will be needed to better understand the relationship between obesity and female reproduction.

## Figures and Tables

**Table 1:** Key adipokines and their functions

Adipokine	Function
TNF 93	Predominantly paracrine, inflammation, insulin resistance and downregulating of anti-inflammatory pathways
Leptin 102	Appetite control and stimulating fatty acid oxidation
Adiponectin 103	Stimulating fatty acid oxidation, reducing gluconeogenesis and anti-inflammatory
Resistin 104	Promoting insulin resistance and inflammation
IL-6 105	Limiting expression of genes encoding inflammatory cytokines and augmenting responsiveness of macrophages to IL-4
IL-8 106	Greater cardiovascular risk
IL-10 107	Anti-inflammatory and immunosuppressive cytokine
IL-18 108	Insulin resistance
Visfatin 109	Enhancing effects of IL-7 and the stem cell factor
Apelin 110	Regulating cardiovascular homeostastis, cell proliferation, food intake, and angiogenesis
MCP-1/CCL2 91	Stimulating macrophage migration
RBP4 111	Insulin resistance

TNF: tumor necrosis factor; IL: interleukin; MCP-1: monocyte chemotactic protein-1; CCL2: Chemokine (C-C motif) ligand 2; RBP4: retinol-binding protein 4.

## Abbreviations

BMI: Body mass index
IVF: *In vitro* fertilization
HPO: Hypothalamic-pituitary-ovarian
LH: Luteinizing hormone
FSH: Follicle stimulating hormone
GnRH: gonadotrophin releasing hormone
GV: Germinal vesicle
GVBD: Germinal vesicle breakdown
HFD: High fat diet
DIO: Diet-induced obesity
ATP: Adenosine triphosphate
TCA: tricarboxylic acid
OXPHOS: oxidative phosphorylation
mtDNA: Mitochondrial DNA
ROS: Reactive oxygen species
ER: Endoplasmic reticulum
COCs: Cumulus-oocyte complexes
DRP1: Dynamin related protein 1
ICM: Inner cell mass
TE: Trophoblast cells
PA: Palmitic acid
LGA: Large for gestational age
DOHaD: Origins of health and disease
MCP-1: Monocyte chemotactic protein-1
LTB4: Leukotriene B4
ATMs: Adipose tissue macrophages
TNF: Tumor necrosis factor
IL: Interleukin
MCP-1: Monocyte chemotactic protein-1
CCL2: Chemokine (C-C motif) ligand 2
RBP4: retinol-binding protein 4
O_2_^-^: Uperoxide anion
^·^OH: Hydroxyl radical
H_2_O_2_: hydrogen peroxide
GC: Granular cell
HATs: Histone acetyl transferases
HDACs: Histone deacetylases
PCOS: Polycystic ovarian syndrome
